# Navigating the doctoral journey: A qualitative study on PhD scholars’ well-being in Pakistan

**DOI:** 10.12669/pjms.41.5.11982

**Published:** 2025-05

**Authors:** Naheed Mahsood, Usman Mahboob, Saima Aleem

**Affiliations:** 1 Naheed Mahsood, Department of Medical Education, Khyber Girls Medical College, Peshawar, Khyber-Pakhtunkhwa, Pakistan; 2 Usman Mahboob, Institute of Health Professions Education & Research, Khyber Medical University, Peshawar, Pakistan; 3 Saima Aleem, Institute of Public Health & Social Sciences, Khyber Medical University, Peshawar, Pakistan

**Keywords:** Doctoral Journey, Higher Education, PhD Scholar Well-being, Qualitative Phenomenology, Socioecological Framework

## Abstract

**Background & Objective:**

Doctor of Philosophy (PhD) scholars face unique challenges during their academic journey, frequently encountering substantial stressors that impact their overall well-being. We aimed to explore the concept and domains of well-being from PhD scholars’ perspectives, focusing on the factors that affect their well-being during doctoral studies.

**Methods:**

This qualitative study was conducted over a period of six months, involving eleven in-depth interviews with medical and allied health sciences PhD scholars from nine universities across Pakistan using a qualitative phenomenology approach. Purposive sampling was employed to recruit participants, and an interview guide was developed after critically appraising the literature, feedback from medical education experts, and a pilot test. Thematic analysis was employed to analyse the data.

**Results:**

Five main themes, accompanied by ten sub-themes, have been developed based on the socioecological theoretical framework, aligning with the key findings from eleven in-depth interviews. Five themes were identified from this study: (i)Well-being at the Individual Level, with the subthemes of mental, physical and emotional well-being and time management and work-life balance, (ii)Well-being at the Interpersonal Level, with the subthemes of supervisor and peer support and family and social support, (iii)Well-being at the Institutional Level, with the subthemes of administrative and financial support and academic stress and resources, (iv)Well-being at the Community Level, with the subthemes of professional growth and networking and cultural and gender influences and (v)Well-being at the Policy Level with the subthemes of HEC and institutional policy gaps and financial and research policy. This multifaceted approach offers a comprehensive understanding of the well-being of PhD scholars by addressing factors at multiple levels.

**Conclusion:**

This study highlighted the multifaceted dimensions of well-being of PhD scholars at individual, interpersonal, institutional, community, and policy levels. PhD scholars’ mental, physical, and emotional health is influenced by time management and academic pressure. PhD scholars’ interpersonal well-being depends on supervisors, peers, and family support. Effective administrative systems, financial support, and academic resources reduce scholar stress and improve institutional well-being. Opportunities for professional growth, networking, and cultural and gender recognition in the community are crucial for improving PhD scholars’ well-being. Our results showed the need for policy reforms that prioritise research-focused PhD programs over coursework-focused ones, create more flexible publication criteria, and ensure enough financial assistance.

## INTRODUCTION

The massification of higher education has broadened the cohort of Doctor of Philosophy (PhD) scholars in medical universities across both developed and developing nations.^1^ PhD scholars are expected to engage in high levels of reasoning while concurrently managing the technical and practical demands of academic work, project management, and production of a doctoral thesis.^2^ The scholarly potential of PhD scholars can be adversely affected by the uncertainty and obstacles they frequently encounter in the academic milieu of doctoral studies.^3^ The well-being of PhD scholars is crucial for the growth of higher education.^4^ Well-being is a complex and multidimensional phenomenon^5^,^6^ hence it is important to assess the well-being of PhD scholars as this population is vulnerable to mental and emotional challenges during their academic journey. There is substantial evidence of distress^7^ and high attrition rates among PhD scholars across a variety of professional fields and countries^8^, leading to intensified concerns about the well-being of PhD scholars in recent years following the documentation of evidence regarding the attrition rate among them.

The majority of evidence originates from Europe, the USA, and Canada, excluding the viewpoints of developing nations.^9^ Well-being is a multifaceted phenomenon, and to comprehend it comprehensively, it is essential to explore the concept of well-being throughout the PhD process, especially in developing countries settings, and its impact on the successful culmination of their academic journey. Moreover, theoretical frameworks have been used previously^4^,^7^, still, a holistic framework such as the social-ecological systems framework can be utilized to fully encompass the concept of well-being. The social-ecological systems framework effectively analyses outcomes within intricate social-ecological systems, presenting a holistic method for comprehending sustainability and governance structures across diverse contexts.^10^ PhD scholars are insufficiently studied and necessitate additional research as a prerequisite for formulating targeted policy designs intended to enhance positive learning settings and programs that support doctoral students.^11^

The aim of our study was to explore the concept and domains of well-being from PhD scholars’ perspectives. This study primarily focused on the PhD scholars enrolled in medical and allied health sciences, given their unique academic and occupational contexts, even though scholars in many disciplines experience varied stressors, PhD scholars in medical and allied health sciences frequently manage stringent research obligations in conjunction with clinical duties, teaching, and patient care, resulting in a multifaceted interaction of pressures^12^ that may vary from those encountered in other fields. This study provides baseline evidence to the administration of universities and policymakers to develop strategies and policies that support PhD scholars’ well-being, aiding them in the timely completion of their PhDs.

## METHODS

An exploratory qualitative study with a phenomenology^13^ approach was conducted over six months, which is useful for gaining an in-depth understanding of the lived experiences of PhD scholars enrolled in different universities of Pakistan in medical and allied health sciences. The study involved eleven doctoral candidates (two males and nine females) from various disciplines. Two participants were from Anatomy, four from Public Health, two from Medical Education, one from Dental Materials, one from Public Health Nutrition, and one from Oral Biology. These disciplines primarily represent basic medical sciences, public health, and dental sciences.

### Ethics approval:

Ethical approval for the study was obtained from the University Ethics Committee of Khyber Medical University (Ref No: 7-1/IHPER/PhD-HPE/KMU/23-04, dated May 24, 2023).

### Participants:

PhD scholars enrolled in medical and allied health science at different universities in Pakistan were invited to participate who have at least completed their coursework of the PhD program to ensure that participants have faced core academic pressures and transitioned from controlled learning to autonomous research, which often presents distinct well-being issues. By this stage, they have experienced academic pressures, supervisory dynamics, and institutional expectations, enabling them to offer detailed insights into the factors affecting their well-being. The purposive sampling technique was used to select the participants for this research.

Of the eleven, three participants were in their second year of PhD studies, focusing on the development of their research synopsis, five participants in the third year engaged in data collection, while three participants in the fourth year focused on data analysis. This distribution facilitated a variety of perspectives on well-being throughout various stages of the PhD journey.

### Procedure:

After a comprehensive literature review, an interview guide was developed, and ten questions covering various aspects of well-being were devised. The guide was finalized after feedback from three medical education experts and a pilot test.

The interview guide was designed as a semi-structured tool to explore key aspects of PhD scholars’ well-being, including the concept and dimensions of well-being, factors affecting well-being, the role of the support system, academic challenges, supervisory relationships, institutional support, higher education policies and coping strategies, as well as suggestions for improving well-being.

The interview guide and consent form were sent to the selected PhD scholars via email/WhatsApp in advance, and their written consent was duly obtained. All individuals participated voluntarily, and confidentiality was maintained by ensuring their anonymity.

In-depth interviews were conducted with eleven PhD scholars enrolled in medical and allied health sciences from nine universities across Pakistan, based on data saturation. The data collection process adhered to the principle of saturation, defined as the point at which no new themes or significant insights arise from further interviews, signifying that adequate data has been obtained to thoroughly comprehend the research phenomenon. Saturation was evaluated via continuous thematic analysis, identifying recurring patterns and concepts across interviews. Upon determining that additional data collection was producing redundancy instead of novel insights, participant recruitment was terminated.

The first author (NM) conducted interviews in both face-to-face and Zoom formats, depending on the scholar’s preference. This hybrid method enabled a comprehensive understanding of the well-being concerns scholars face in various contexts across Pakistan. Only the participant and researcher were present during the interview to ensure privacy and minimize external influence on the data collection process.

Each interview lasted between 40 and 50 minutes, and probing questions were employed as necessary to acquire a more profound understanding of the participants’ experiences. To maintain confidentiality, each scholar was allocated a distinct code name (e.g., Participant A, Participant B) in the order of the interviews. All interviews were audio recorded and transcribed verbatim.

### Data analysis:

The data were analysed manually with an inductive approach. The transcribed data, written notes, and audio recordings were meticulously reviewed to enhance familiarity with the topic. Initial codes were allocated to key elements in the raw data to enable meaningful interpretation. The codes were further reviewed and categorized, with analogous codes consolidated to establish potential themes or sub-themes. Following a second round of coding, sub-themes were generated. Finally, we analysed how each sub-theme encapsulated particular facets of participants’ experiences pertinent to the research topic.

A team-based strategy was utilised to maintain uniformity and reduce discrepancies in data coding and theme identification. Initially, two researchers separately analysed a selection of the data to discern preliminary themes. Discrepancies among the coders were addressed in regular sessions, during which the research team collaboratively examined and improved the codes as necessary. An iterative procedure was employed, wherein codes and themes were examined and refined throughout the investigation to ensure coherence and consistency. A conclusive agreement on the identified themes was attained through collaborative discussion and verification.

### Quality assurance:

To ensure trustworthiness and rigor in our study, we addressed four core criteria of quality in qualitative research, i.e. credibility, transferability, dependability, and confirmability.^13^ We ensured credibility through triangulation and member-checking. Peer debriefing was employed to facilitate triangulation. The authors thoroughly examined interview transcripts and audio recordings. Each PhD scholar was provided with their transcribed interview for review, and only after their approval was data further analysed. Dependability was established through comprehensive documentation of each step of the research process. To ensure transferability, the study included a detailed description of the phenomena, including thorough narratives from interviews and direct quotations from participants. Confirmability was enhanced by sustaining a comprehensive audit trail and meticulously documenting code decisions, data analysis procedures, and iterations of developing coding schemes. The Consolidated Criteria for Reporting Qualitative Research (COREQ-32) checklist was utilized to ensure the quality of exploratory studies assessing complex phenomena.

## RESULTS

**Fig.1:**
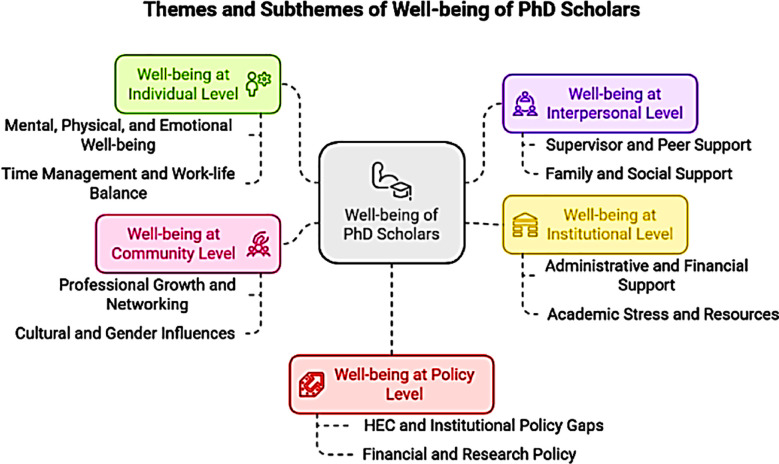
Themes and Subthemes of Well-being of PhD Students.

Thematic analysis was done to create meaningful patterns through the process of coding in six phases, i.e., familiarization with data, generating initial codes, searching for themes among codes, reviewing themes, defining and naming themes, and producing the final report.^14^ Five themes have been developed based on the socioecological theoretical framework at the individual, interpersonal, institutional, community, and policy levels. Five main themes were accompanied by ten supporting sub-themes (Fig.1) and sixty-nine codes in the final analysis (Table-I).

**Table-I:** Themes, Subthemes, Codes, and Quotes related to Well-being of PhD Scholars

Themes	Sub-themes	Codes	Quotes
1. Well-being at the Individual Level	Mental, Physical, and Emotional Well-being	Stress, Burnout, Physical health decline, Emotional rollercoaster, Emotional exhaustion, Mental and emotional impact, Emotional instability, Mental resilience, Physical health concerns	*"I felt burnt out after the comprehensive exam. Physical, mental, and emotional health are interconnected and affected during a PhD"* (**Participant J**)
	Time Management and Work-Life Balance	Juggling multiple roles, Time management, balancing responsibilities, stress management, Parental responsibilities, Work-life balance struggles	*"I work from 10 am till 3 pm daily, and after that, it’s my family time. Balancing PhD, job, and family is a constant challenge."* (**Participant A**)
2. Well-being at the Interpersonal Level	Supervisor and Peer Support	Supervisor compatibility, Colleague support, Peer competition, Supervisory challenges, Lack of supervisor availability, Supervisor accountability, Negative supervisor influence, Delayed feedback issues	*"Supervisor’s availability is crucial to my well-being."* (**Participant H**) *"Peer support helps you a lot, I used to call my peers when something was not clear to me."* (**Participant J**)
	Family and Social Support	Family support, Social connections	*“My family has been exceptionally supportive.” “Social connections helped me access research materials.”* (**Participant B**)
3. Well-being at the Institutional Level	Administrative and Financial Support	Institutional support, Administrative pressure, Institutional delays, Nonresponsive administration, Insufficient Institutional Support, Financial stress, Limited institutional funding, Institutional dissatisfaction, Administrative challenges, lack of Institutional financial support, Inadequate research funding, Financial challenges,	*"The university provides very limited funds for our PhD research project."* (**Participant K**) *"Institutional support has a significant role, but unfortunately, I didn’t receive any help from the institution."* (**Participant J**)
	Academic Stress and Resources	Inadequate research resources, Deficient lab equipment, Inadequate infrastructure, Academic workload stress, Stress from exams, Academic pressure, Burnout from coursework, Overlaps of professional duties with academic activities, Stress from academic overload, Research culture challenges, Need for university resources, Improved resources, Research facility needs	*“We are pushed to our limits by the intense academic pressure of PhD coursework and comprehensive exams."* (**Participant F**) *"There is no proper space for seating and no access to proper databases for our assignments."* (**Participant E**)
4. Well-being at the Community Level	Professional Growth and Networking	Social network, Scholarly community, Social connections, Social isolation	*“Social contacts helped me access research materials and articles.” “My PR increases, and my knowledge expands through social connections.”* (**Participant A**)
	Cultural and Gender Influences	Gender roles, Cultural norms, Culture of favoritism, Gender-related stressors, Gender disparities, Social construct of gender	*“As a female… balancing PhD studies tasks with your other responsibilities makes your life stressful.”* (**Participant G**)
5. Well-being at the Policy Level	HEC and Institutional Policy Gaps	Policy challenges, Administrative delays, Need for scholar-friendly policies, Simplify PhD process, Research-based coursework policy, Policy and program reform	*“HEC policies should be scholar-friendly, not system-friendly.”* (**Participant H**)
	Financial and Research Policy	Lack of funding, research-focused reforms	*“HEC policies impact our well-being… A PhD is a research-based program that should prioritize research… PhD programs should focus on research rather than mandatory coursework."* (**Participant I**)

Participants who had recently completed their coursework and were engaged in developing their synopses primarily identified challenges associated with the transition from structured coursework to independent research. These challenges included uncertainty regarding research direction, database access for literature review, and difficulties in proposal development. Conversely, individuals involved in the data collection and analysis phases experienced increased stress associated with managing research logistics, recruiting participants, and handling extensive datasets. PhD scholars in the final phases highlighted difficulties associated with writing, publishing, and adhering to institutional deadlines. This variation in experiences highlights the evolution of well-being concerns during the PhD journey, emphasising the necessity for ongoing institutional and supervisory support at various stages of doctoral research.

### Theme-1: Well-being of PhD scholars at the Individual Level

### Subtheme: Mental, Physical, and Emotional Well-being:

Mental, physical, and emotional well-being were essential components of a PhD scholar’s overall well-being, each vital for cultivating resilience in adversity. PhD scholars endured considerable mental, emotional, and physical stress, frequently encountering obstacles such as anxiety, emotional turmoil, and health issues due to their workload.

*“I have been affected mentally, physically, and emotionally. I felt exhausted and sleep-deprived, I have anorexia-type symptoms.” **(Participant D)***

### Subtheme: Time Management and Work-life Balance:

Time management and work-life balance pose significant obstacles for PhD scholars, whose academic pursuits are frequently characterized by rigorous demands and minimal structure. Managing coursework, research obligations, publication efforts, and sometimes teaching, in conjunction with job and personal commitments, can generate significant stress. Effective time management is crucial, as scholars must allocate time to fulfill academic requirements, pursue professional development, and sustain personal well-being.

*“Balancing PhD studies tasks with your personal and professional responsibilities makes your life stressful.”**(Participant G)***

### Theme-2: Well-being of PhD scholars at the Interpersonal Level

### Subthemes: Supervisor and Peer Support:

Supervisor and peer support are essential for PhD scholars, influencing both academic achievement and overall well-being. A supportive supervisor offers guidance, constructive feedback, and motivation, assisting academics in overcoming research obstacles. Peer support provides companionship and mutual understanding, mitigating isolation through collaborative discussion and resource sharing. Collectively, these support structures cultivate drive, resilience, and a balanced academic experience.

*“Supervisors have a very important role in motivating you. Peer support is also very crucial. My peers gave me lots of support during coursework.” **(Participant F)***

### Subtheme: Family and Social Support:

Family and social support are crucial for PhD scholars, offering stability and resilience in the face of academic pressures. Family supports and encourages, while friends and social networks allow leisure and stress alleviation. Collectively, these support systems augment motivation, reduce stress, and promote well-being

*“I am from an academic background, and my family and friends provided a lot of support.”**(Participant B)***

### Theme 3: Well-being of PhD scholars at the Institutional Level

### Subtheme: Administrative and Financial Support:

Administrative and financial support are essential for enhancing the well-being of PhD scholars at the institutional level. Administrative support, encompassing streamlined procedures for course registration, prompt responses, and transparent communication of requirements, alleviates the administrative strain on scholars. Financial support, via scholarships, research grants, and stipends, directly influences scholars’ capacity to handle living expenses, participate in conferences, and obtain vital resources, alleviating financial strain. Collectively, these support mechanisms foster an atmosphere in which scholars see institutional worth, so enhancing their capacity to sustain well-being.

*“Finances are a big issue. My main concern is the cost of research materials… it becomes very stressful for a PhD scholar. The university provides very limited funds for our PhD research project.”**(Participant A)***

### Subtheme: Academic Stress and Resources:

Academic stress for PhD scholars frequently arises from stringent course requirements, exhaustive assessments, and the expectation for substantial research productivity. Achieving these scholastic milestones necessitates extensive preparation, hence increasing stress levels and affecting the well-being of PhD scholars. Access to sufficient institutional resources is crucial for addressing these challenges. Supplying research resources, equipment, and database access enhances research efficacy, allowing scholars to achieve their academic objectives.

*“I faced lots of academic workloads, especially the back-to-back assessments… very stressful and time-consuming. Our labs are very deficient, and we have to purchase most materials ourselves. The lack of resources makes the PhD process more stressful.”**(Participant J)***

### Theme-4: Well-being of PhD scholars at the Community Level

### Subtheme: Professional Growth and Networking:

PhD scholars’ well-being is significantly influenced by professional growth and networking, which cultivate resilience and academic success. Positive social networks and opportunities to engage with scholarly communities reduce feelings of social isolation. Being part of a supportive scholarly community provides access to valuable resources and beneficial opportunities that enhance visibility and knowledge in the field positively impacting their overall well-being.

*“If you are socially connected with the scholarly community, it will be helpful for your research project.” **(Participant C)***

### Subtheme: Cultural and Gender Influences:

The well-being of PhD scholars is significantly influenced by cultural and gender factors, particularly in environments where traditional norms and expectations define academic experiences. Gender-based responsibilities, such as family caregiving, frequently result in significant tension for female scholars, who may find it difficult to reconcile academic demands with societal expectations. Furthermore, cultural issues, e.g., language differences, varying social norms, and gender disparities in academic environments, can establish barriers, causing certain scholars to feel overlooked or unsupported as a result of biases, and they may even withdraw from their programs.

*“I believe that it’s difficult for Female scholars to pursue their PhDs with small kids.”*

*“I faced a language barrier initially as I was from a different province and unfamiliar with the local language.” **(Participant F)***

### Theme-5: Well-being of PhD scholars at the Policy Level

### Subtheme: HEC and Institutional Policy Gaps:

Assessing the well-being of PhD scholars at the policy level reveals substantial deficiencies in the Higher Education Commission (HEC) and institutional policies, especially those affecting the academic experience of scholars. Current PhD policies, particularly those emphasizing extensive coursework, are often recognized as significant stressors, indicating a necessity for a transition to more research-focused coursework policies. Administrative delays and policy obstacles exacerbate stress, prompting experts to advocate for streamlined procedures and diminished bureaucratic impediments to enhance efficiency. A distinct demand exists for scholar-centric policies that streamline the PhD process, enabling researchers to concentrate more efficiently on their research endeavours.

*“PhD program should be research-based, there is no need for coursework and comprehensive exams, as it gives you lots of stress and pressure, and I find it a very weird liability” **(Participant F)***

### Subtheme: Financial and Research Policy:

The subtheme of Financial and Research Policy emphasizes the necessity for more financing and research-oriented reforms to enhance the well-being of PhD scholars. Researchers often encounter financial constraints that impede research activities and publication endeavours, especially due to the substantial expenses linked to publishing in high-impact factor journals.

*“The publication policy of HEC is another big issue, the obligation of publication in a high impact factor needs lots of finances, which HEC or the university doesn’t give you.” **(Participant H)***

## DISCUSSION

The well-being of PhD scholars is a multifaceted and multidimensional phenomenon shaped by individual, interpersonal, institutional, community, and policy-level factors.

At the individual level, the physical, mental, and emotional health of the scholar is usually compromised due to the arduous demands of doctoral studies. They face challenges of maintaining work-life balance and time management, which ultimately result in exhaustion and burnout. Levecque reported similar findings that PhD scholars face a higher risk of mental health issues than the general population, and the primary stressors are academic pressure and insufficient time.^15^ This study also identified similar findings of work-life balance and time management issues affecting the well-being of PhD scholars.

At the Interpersonal level, various factors affect the well-being of PhD scholars, especially their relationships with supervisors, peers, and family. Positive collaboration with supervisors and peers is associated with better academic performance and personal fulfillment. Their ability to cope with stress and anxiety is improved if they get support from family and friends. In contrast, insufficient social support can intensify stress and feelings of isolation. Maha Al Makhamreh reported the importance of supportive supervision, emphasizing that supportive supervision can reduce stress and enhance the academic achievement of scholars.^16^ Various studies highlighted the importance of supervisor support in promoting the resilience and well-being of PhD scholars.^17^-^19^ However, a few of our study participants who have received strong peer support, Castelló found that competitive or isolated environments in some academic settings might impede peer interaction, leading to feelings of isolation and loneliness among scholars.^20^ The significance of social support, especially supervisor and peer support, was highlighted in a study as an essential element for the well-being of doctoral students and potentially mitigating the risk of dropout.^21^

At the institutional level, the well-being of PhD scholars is affected by administrative efficiency, financial assistance, and access to resources. Any sort of administrative delays, lack of funding, and limited access to resources can hinder the progress of the scholar. Obstacles at the institutional level, especially administrative delays and financial issues, have been reported in previous studies. Pyhältö reported that administrative delays and financial constraints are considerable stressors for the PhD scholars, impeding their research progress and overall well-being.^22^ Several studies highlighted the significance of financial support for PhD scholars, enabling them to focus on academic achievement and reducing their stress.^23^-^25^ For institutions to compete at a global level, it is of utmost importance for their researchers and faculty to achieve higher academic titles and considering a PhD as a terminal qualification, institutional support is the backbone of a successful doctorate degree.^26^ This study result emphasized the importance of administrative efficiency and the provision of adequate financial support for PhD scholars at the institutional level.

At the Community level, PhD scholars’ long-term career fulfillment depends on the opportunities for professional growth and networking. Eleonora Cilli reported that professional networking significantly enhances the resilience and career satisfaction of scholars, emphasizing the importance of professional growth and networking opportunities.^27^

The well-being of the scholar is also affected by the cultural expectations and social construction of gender roles, as some scholars face distinctive obstacles associated with their gender roles in society. It was reported in a study that there is a complex interplay between cultural norms and academic environment, emphasizing that factors such as race, gender, age, and educational background of the students can affect their academic experience during their transition to autonomy, indicating that further research is needed to analyse these processes more comprehensively.^28^ Aitchison and Mowbray investigated the challenges faced by women PhD scholars stemming from societal expectations and conventional gender norms, highlighting that stress from academic and family responsibilities, emotional labor, and isolation affect the well-being of women scholars, and they need a support network, including counselling and mindfulness practices.^29^

Finally, well-being at the policy level revealed deficiencies in both HEC and institutional policies, including the necessity for increased financing and less coursework in PhD programs. Current policies prioritizing high-impact factor publications put the academic and financial burden on PhD scholars.^30^ Implementing reforms to address these deficiencies could more effectively match program structures with the requirements of researchers, thus boosting their well-being and elevating the quality of PhD education. The publication requirements and coursework-heavy PhD structures are consistent with previous research. Sverdlik reported that many PhD programs could benefit from policy reform that is more research-focused, rather than coursework-focused programs, prioritizing scholars’ autonomy and mitigating unnecessary stress.^9^ The findings of this study corroborate their results by indicating that policies mandating high-impact factor articles often impose excessive stress on scholars, hence requiring more accommodating and supportive publication policies.

The results highlighted the obstacles encountered by PhD scholars in various contexts and the necessity for targeted reforms at the personal, interpersonal, institutional, community, and policy levels to improve the well-being of PhD scholars. To implement the proposed policy reforms, institutions could augment administrative assistance by forming specialised teams to aid PhD scholars with research-related activities and improving access to research resources. The Higher Education Commission (HEC) might enhance PhD funding by providing additional scholarships and research grants, as well as establishing more explicit research requirements. Moreover, PhD supervisors must be urged to adhere to standardised mentorship protocols, encompassing regular progress meetings, tailored support, and fostering professional development opportunities. The strategies presented in this study provide concrete measures to enhance the PhD experience and well-being of scholars.

### Limitations:

This study has some limitations, as the findings depend on the experience of study participants, which may not reflect the diversity of obstacles faced by PhD scholars in various academic disciplines or geographic regions. Additionally, the responses of participants may be influenced by potential biases in self-reporting, as certain scholars may either overstate or understate their experiences. Lastly, the study emphasizes the perspectives of currently enrolled scholars, neglecting the perspectives of those who have completed or discontinued their PhDs, who may offer valuable insights into the long-term well-being of PhD scholars.

## CONCLUSION

This study emphasized the multifaceted dimensions of the well-being of PhD scholars at individual, interpersonal, institutional, community, and policy levels, emphasizing support at each level. The results highlighted the mental, physical, and emotional issues of PhD scholars due to time management and excessive academic pressure, affecting the well-being of PhD scholars at the individual level. It is emphasized that support from supervisors, peers, and family is crucial for the well-being of PhD scholars at the interpersonal level. Efficient administrative processes, financial support, and access to academic resources are essential to mitigate scholars’ stress and enhance their well-being at the institutional level. Moreover, the opportunities for professional growth and networking, and recognition of cultural and gender dynamics within the community are essential for cultivating a strong academic atmosphere and enhancing the well-being of PhD scholars at the community level. This study also highlighted the need for policy reforms that focus on prioritizing research-focused PhD programs instead of heavy coursework-focused PhD programs, developing more flexible and accommodating publication guidelines, and guaranteeing adequate financial support. Addressing these issues is crucial for fostering an academic environment that supports the well-being of PhD scholars through informed policy initiatives.

### Recommendations:

Future research direction may include longitudinal studies to assess the well-being of PhD scholars over the course of their program, or conduct cross-cultural comparative studies, or discipline-specific research for scholars in fields other than medical and allied health sciences to explore the impact of contextual factors on the well-being of PhD scholars in various academic settings.

### Data availability:

The dataset supporting this study’s findings is available upon request from the corresponding author (UM). Nevertheless, the data remain inaccessible to the public due to restrictions, including the potential jeopardizing of research participants’ privacy.
